# Cancer care in times of conflict: Treatment of patients from Afghanistan, in Pakistan

**DOI:** 10.3389/fonc.2023.1110709

**Published:** 2023-03-03

**Authors:** Shahid Mahmood, Shehryar Nasir Khawaja, Muhammed Aasim Yusuf

**Affiliations:** ^1^ Department of Cancer Registry and Clinical Data Management, Shaukat Khanum Memorial Cancer Hospital and Research Centres, Lahore, Pakistan; ^2^ Department of Internal Medicine, Shaukat Khanum Memorial Cancer Hospital and Research Centres, Lahore, Pakistan

**Keywords:** Afghanistan, cancer, cancer management, cancer registry, conflict

## Abstract

**Introduction:**

Afghanistan has been in a near-continuous armed conflict, which has degraded the country’s health infrastructure. Due to this, Afghans have opted to seek cancer treatment in Pakistan. This manuscript aims to understand the complex cancer journey of patients from Afghanistan seeking care in the largest tertiary care cancer institutions in Pakistan.

**Methods:**

This retrospective study explores the demographics, epidemiology and outcomes of Afghan cancer patients treated at the Shaukat Khanum Memorial Cancer Hospital and Research Centres (SKMCH&RC) in Lahore and Peshawar, Pakistan, over the period from 1995 to June 2022.

**Results:**

A total of 6,370 patients from Afghanistan have undergone cancer care since 1995. The mean age at presentation was 40.7 years, 57% were male, and 87% were adults >19 years. Close to 30% of these patients came from Kabul and Nangarhar districts. 56% of all patients presented with stage III or IV disease. 34% of adult patients achieved a complete response to treatment, but more than half of all patients have since been lost to follow-up. Children generally had better outcomes, with 43% showing a complete response to treatment.

**Discussion:**

The cancer journey for these patients remains long and difficult and the inability to ensure follow-up in so many remains frustrating for both patients and providers. The lack of a cancer infrastructure in Afghanistan after decades of conflict, means that policymakers need to develop and support alternative systems and structures to provide post-conflict domestic and cross-border cancer care.

## Introduction

Worldwide, 89.3 million people are currently forcibly displaced as a result of persecution, conflict, and other events disturbing public order. Low- and middle-income countries host 83% of these individuals. By the end of 2021, approximately 2.7 million Afghans were displaced across borders, and an estimated 3.5 million were displaced internally. These figures have risen for each of the preceding fifteen years (1). Armed conflicts have short and long-term socio-economic, geopolitical, and health consequences and the international community has repeatedly responded to refugee crises, mostly through United Nations (UN) agencies and non-governmental organisations (NGOs) (2). These efforts, however, have focused primarily on providing acute medical care, or on controlling public health issues such as malnutrition and outbreaks of infectious disease (3). Non-communicable diseases, especially cancer, in refugee and migrant populations have received much less international attention. Refugee and migrant populations often present with advanced disease, are unfamiliar with health systems in host countries and are not usually enrolled in formal care programmes, all of which make it more likely that they will have poor health outcomes (3–5). 

Pakistan shares a long border with Afghanistan. Following the invasion of Afghanistan by the former Union of Soviet Socialist Republics (USSR) in 1979, millions of people from Afghanistan crossed the border to seek refuge in Pakistan. At its peak, the refugee population within Pakistan numbered over 3.3 million people (1, 3).

The Shaukat Khanum Memorial Cancer Hospital and Research Centre (SKMCH&RC) was set up in Lahore, Pakistan in 1994. The intake mechanism for cancer patients is based on a diagnosis of cancer, without regard to financial status, ethnicity, or nationality. Nearly 30% of all patients seen in the hospital in Lahore have been from the north-western province of Pakistan, bordering Afghanistan, called Khyber Pakhtunkhwa (KP), or from Afghanistan. Recognising the need for a specialised tertiary care cancer hospital in KP, a second SKMCH&RC was established in Peshawar, the provincial capital, in 2015.

We have previously published data regarding the clinical, geo- and socio-demographic features of refugees from Afghanistan who sought care at SKMCH&RC (3). However, since then, the geopolitical landscape of Afghanistan has changed, and SKMCH&RC has opened another hospital near the Afghanistan border in Peshawar, which has increased the number of patients receiving care. Hence, we present an updated retrospective analysis to help understand the complex cancer journey of patients from Afghanistan seeking care in the largest tertiary care cancer institutions in Pakistan.

## Materials and methods

A retrospective review of patients identified as Afghan nationals, or patients who provided an address in Afghanistan at the time of initial registration at Shaukat Khanum Memorial Cancer Hospital & Research Centre (SKMCH&RC), between the 1st of December 1995 and the 30th of June 2022 was performed. From this cohort, all male and female patients who received treatment for cancer were evaluated further. Patients with benign aetiology or those who did not complete diagnostic work-up were excluded from further analysis.

One of the core principles of SKMCH&RC, enshrined in its mission statement, is to provide the best possible cancer treatment to all our patients, irrespective of their ability to pay. SKMCH&RC has Walk-In clinics across the country, where patients with an established cancer diagnosis, as well as those with a suspicion of cancer, may come to be assessed for acceptance into the system. It is estimated that there are close to 180,000 new cancer diagnoses each year in Pakistan alone and of these, close to 45,000 patients come to one of SKMCH&RC Walk-In clinics, seeking treatment, each year.

At SKMCH&RC, most chemotherapy and all radiation treatments are day-case or outpatient procedures. Almost all patients need a place to stay near one of our two hospitals for the duration of their treatment and while some may stay in small hotels which have sprung up nearby, most cannot afford even this Spartan accommodation. To cater to the needs of such individuals, supporters of the Hospital have provided access to nearby hostel facilities, or Musafirkhanas, for three hundred patients, where free accommodation and meals are provided to these patients as well as to one carer each. A significant proportion of the patients who access these facilities are Afghans or Pakistani Pashtun patients from KP, and this has led to the development of an environment where Pashtu and Farsi (Persian) are spoken, and where an informal support system has grown organically, helping to ameliorate the difficulties these patients experience at a difficult time and in a somewhat alien environment.

Since we cannot treat all the cancer patients who come to us, we accept patients based on diagnosis and cancer stage, the intention being to accept those most likely to be curable. Nonetheless, once patients are accepted for treatment, they are eligible for all treatments available, even when their cancers progress, regardless of their ability to pay and irrespective of their nationality or ethnicity.

SKMCH&RC uses a custom-built electronic medical record system consisting of patient registration, clinical information, order entry, and results viewing modules. The system was commissioned in 2000, before which the medical record was paper based. Older, paper-based records have since been scanned, archived, and integrated into the electronic system.

The data for all participants was de-identified, and patient records were reviewed to gather data on patient demographics, including address within Afghanistan, gender, cancer diagnosis, stage, treatment provided and follow-up details. The descriptive analysis was conducted on Microsoft Excel.

## Results

A total of 113,384 patients were registered for cancer care between December 1995 and June 2022 at SKMCH&RC. Among these, 7,468 (6.6%) patients were identified as Afghan nationals. The records of 1,098 (14.7%) patients were excluded, as they did not receive treatment at SKMCH&RC due to benign aetiology, because they did not return after an initial visit or because they failed to complete staging investigations.

**Figure 1:**
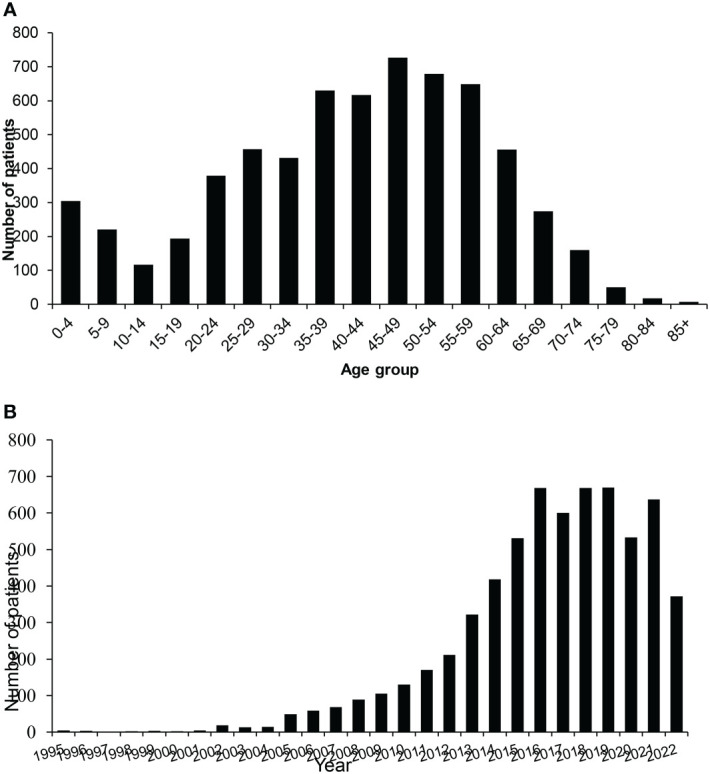
Afghan migrants treated at SKMCH&RC (n = 6,370) from 1995 to 30^th^ June 2022. **(A)** Age distribution. **(B)** Frequency of Afghan patients by year.

Of the 6,370 patients included in this study, 57% were male. The mean age at presentation was 40.7 years and 87% of the participants were adults (**Figure 1A**). There was a dramatic increase in Afghan migrants registering for care from 2012 onwards, peaking in 2018, despite relative peace and stability, at least in some parts of Afghanistan, during this period (**Figure 1B**). Most patients were born in Afghanistan (92%), and a slightly lower percentage had an active Afghanistan address (83%). The largest number of patients came from Kabul (19%) followed by Nangarhar (11%), Herat (6%), Balkh (5%) and Ghazni (4%). Each of the other provinces accounted for less than 5% of the patients seen. The province of origin for 16% of patients was unknown.

Over 56% of all patients (56% of adults and 41% of children) presented with advanced disease (Stage III and IV), while cancer stage was not documented in 16%. Details of cancer stages of the participants are given in **Table 1**. 54% of patients were lost to follow-up, 23% of patients were being actively followed-up at the time of data extraction, and 8% were known to have died. These results are stratified in **Table 2**.

**Table 1:** Stage distribution among paediatric, adult, and total patients.

	No. (%)		
	Paediatrics (n = 836)	Adult (n = 5,534)	Total (n = 6,370)
**Stage 0**	0 (0.0)	24 (0.4)	24 (0.4)
**Stage I**	122 (14.6)	525 (9.5)	647 (10.2)
**Stage II**	96 (11.5)	1,101 (19.9)	1,197 (18.8)
**Stage III**	199 (23.8)	2,047 (37.0)	2,246 (35.3)
**Stage IV**	151 (18.1)	1061 (19.2)	1212 (19.0)
**Unknown/Not applicable**	268 (32.1)	776 (14.0)	1044 (16.4)

**Table 2:** Distribution of select outcomes among paediatric, adult, and total patients.

Select outcomes	No. (%)		
	Paediatrics (n = 836)	Adult (n = 5,534)	Total (n = 6,370)
**Dead**	126 (15.1)	398 (7.2)	524 (8.2)
**Discharged**	56 (6.7)	227 (4.1)	283 (4.4)
**Lost to follow-up**	325 (38.9)	3,142 (56.8)	3,467 (54.4)
**On follow-up**	215 (25.7)	1,244 (22.5)	1,459 (22.9)
**On treatment**	114 (13.6)	523 (9.5)	637 (10.0)

**Figure 2:**
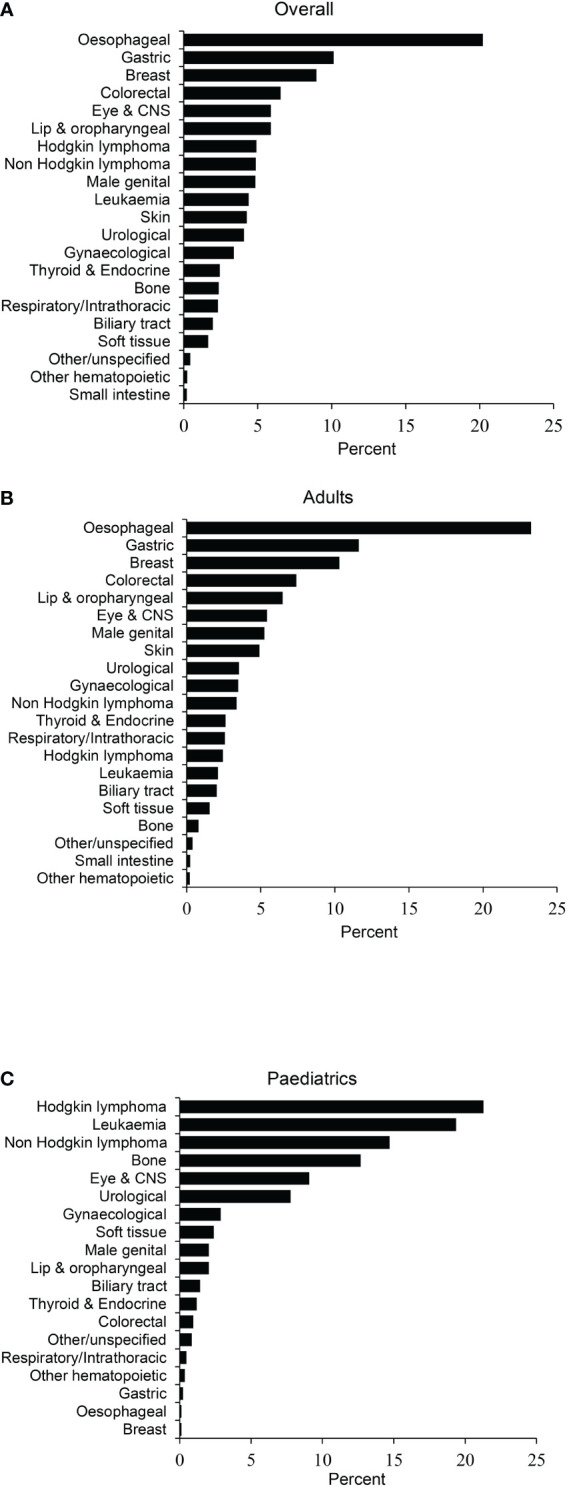
Distribution by site. **(A)** Overall, **(B)** Adult, and **(C)** Childhood cancers. ALL, Acute lymphoblastic leukaemia; CML, chronic myeloid leukaemia; CNS, Central Nervous System; NHL, non-Hodgkin lymphoma; AML, Acute myeloid leukaemia.

Upper gastrointestinal malignancies including oesophageal (20%) and gastric cancer (10%) were the most common cancers among adult patients, followed by breast (10.1%) and colorectal cancer (7%). Haematological malignancies including Hodgkin lymphoma (21%), acute lymphoblastic leukaemia (17%) and non-Hodgkin lymphoma (14%), were the most common cancers in paediatric cases (**Figure 2**).

The most common cancers among males were oesophageal (17.2%), gastric (13.7%), and colorectal. In females, by contrast, the commonest cancers seen were of the oesophagus (24.2%) and breast (20.6%) (**Table 3**).

**Table 3:** Most frequent primary cancers by gender (n= 6,370).

S. No	Primary Site	Female (n = 2,742)		Male (n = 3,628)		Total (n = 6,370)	
		Cases	%	Cases	%	Cases	%
1	Esophagus	663	24.2%	625	17.2%	1288	20.2%
2	Stomach	148	5.4%	497	13.7%	645	10.1%
3	Breast	566	20.6%	5	0.1%	571	9.0%
4	Colorectal	131	4.8%	287	7.9%	418	6.6%
5	Hodgkin lymphoma	84	3.1%	229	6.3%	313	4.9%
6	Non-Hodgkin lymphoma	92	3.4%	218	6.0%	310	4.9%
7	Leukemia	94	3.4%	185	5.1%	279	4.4%
8	Skin	83	3.0%	190	5.2%	273	4.3%
9	Brain and spinal cord	94	3.4%	172	4.7%	266	4.1%
10	Testis	0	0.0%	182	5.0%	182	2.9%
11	Kidney	71	2.6%	105	2.9%	176	2.8%
12	Lip and oral cavity	66	2.4%	110	3.0%	176	2.8%
13	Bone	48	1.8%	102	2.8%	150	2.4%
14	Nasopharynx	37	1.3%	98	2.7%	135	2.1%
15	Thyroid	88	3.2%	42	1.2%	130	2.0%
16	Prostate	0	0.0%	125	3.4%	125	2.0%
17	Cervix uteri	112	4.1%	0	0.0%	112	1.8%
18	Soft tissues	46	1.7%	59	1.6%	105	1.6%
19	Urinary bladder	20	0.7%	63	1.7%	83	1.3%
20	Gallbladder & extrahepatic bile ducts	32	1.2%	36	1.0%	68	1.1%
21	Ovary and uterine adnexa	61	2.2%	0	0.0%	61	1.0%
22	Trachea, bronchus & lung	18	0.7%	38	1.0%	56	0.9%
23	Eye and adnexa	23	0.8%	31	0.9%	54	0.8%
24	Hypopharynx	20	0.7%	32	0.9%	52	0.8%
25	Larynx	2	0.1%	41	1.1%	43	0.7%
26	Nasal cavity and accessory sinuses	14	0.5%	22	0.6%	36	0.6%
27	Corpus uteri and uterus, NOS	35	1.3%	0	0.0%	35	0.5%
28	Pancreas	14	0.5%	21	0.6%	35	0.5%
29	Liver and intrahepatic bile ducts	10	0.4%	12	0.3%	22	0.3%
30	Other miscellaneous sites	70	2.6%	101	2.8%	171	2.7%

**Figure 3:**
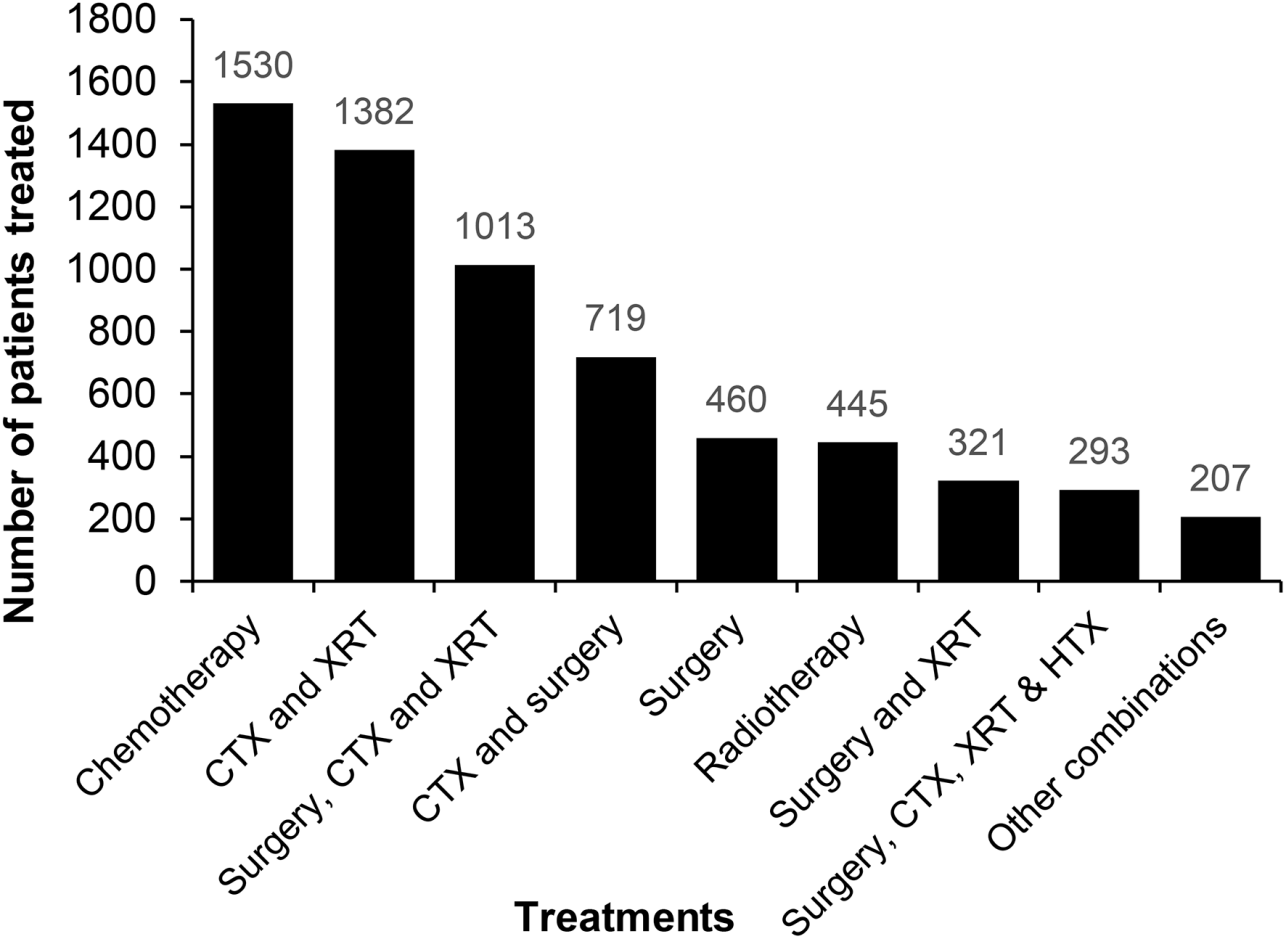
Treatment outcomes for adult male and female patients.

Patients received a range of treatments, in keeping with the heterogeneity of cancer diagnoses. Nearly three-quarters of patients were treated with chemotherapy (CTX) or a combination of chemotherapy and external beam radiation (XRT), with or without surgery. Other treatments provided included surgery, alone or in combination with other treatment modalities, in 44%, hormone therapy (HTX) and immunotherapy (**Figure 3**). Amongst adult patients, 34% achieved a complete response, 10% had a partial response, 8% had stable disease and 29% relapsed or had progressive disease. We were unable to assess disease outcome in 19% of patients. 43% of paediatric patients achieved a complete response to therapy, 12% had a partial response, 3% had stable disease and 16% relapsed or had progressive disease. We were unable to assess disease outcome in 26% of paediatric patients. More than two-thirds of paediatric patients were treated with CTX alone, in keeping with the types of cancer diagnoses (**Table 4**).

**Table 4:** Distribution of treatment responses among paediatric, adult, and total patients.

Select outcomes	No. (%)		
	Paediatrics (n = 836)	Adult (n = 5,534)	Total (n = 6,370)
**Complete response**	355 (42.5)	1,906 (34.4)	2,261 (35.5)
**Partial response**	103 (12.3)	541 (9.8)	644 (10.1)
**Stable disease**	27 (3.2)	423 (7.6)	450 (7.1)
**Progression/relapse**	136 (16.3)	1593 (28.8)	1,729 (27.1)
**Unknown**	215 (25.7)	1071 (19.4)	1,286 (20.2)

## Discussion

Most patients who presented to SKMCH&RC hospitals from Afghanistan were treatment-naïve, but the majority had had some initial work-up, including a biopsy to establish a cancer diagnosis, in their own country. While a small number had had an attempt at curative surgery, very few had received chemotherapy, and none had radiation therapy, since there are currently no radiation-treatment facilities in Afghanistan.

More than half of the patients we saw from Afghanistan presented with advanced-stage disease. We postulate that there may be many reasons for this, including the taboo associated with cancer, poor health quotient, the lack of local health care facilities, low socioeconomic status, hesitancy to seek care, or because of traveling difficulties involved in seeking care overseas (6, 7). While many patients presented from parts of Afghanistan close to, or contiguous with Pakistan, others travelled up to 2,000 km or for up to 26 hours, in order to seek cancer treatment at our hospitals (3). The poor travel infrastructure, compounded by the perilous security situation, make these journeys extremely arduous (8). Half of all patients were lost to follow-up following initial definitive treatment, undoubtedly due, at least in part, to travel and visa obstacles. All Afghan patients coming to Pakistan do so on a visa. Typically, visas are issued for periods of 2 to 4 weeks at a time, and patients undergoing prolonged cancer treatment need to go back and forth in order to renew their visas. As a result, many miss important appointments for investigations or treatment, and the difficulties involved in this process undoubtedly dampen enthusiasm to return for follow up appointments, once treatment has been completed.

The most common malignancy among adults in the present study cohort were upper gastrointestinal malignancies, including oesophageal and gastric cancers, followed by breast and colorectal cancer. Oesophageal and gastric cancers were the most common cancers among males, and oesophageal and breast tumours had the highest incidence among females. Similar results among Afghans have been reported by others (9, 10). Interestingly, these numbers are comparable to the cancer statistics among Pakistanis and Iranians (10–12). A recent study of Afghan oesophageal cancer patients has suggested that living in a rural area, illiteracy, consuming oral snuff, drinking hot tea, lack of physical exercise, modest consumption of fruit, and positive family history of cancer were significantly associated with the development of oesophageal cancer (13).

Similarly, among the paediatric population, haematological malignancies, including Hodgkin lymphoma, acute lymphoblastic leukaemia, and non-Hodgkin lymphoma, were common. The literature on the incidence of malignancies among the paediatric population in Afghanistan is relatively scarce and ambiguous (10, 13–15). Nonetheless, prior investigations have shown that lymphoma and acute lymphoblastic leukaemia are the most common haematological malignancies seen in the Afghan paediatric population (14, 15).

In the present study, patients received a wide array of oncological therapies in different combinations. The complete response rate was less than 50%, with a lower response rate in adults than in the paediatric population, with all rates being lower than those reported in high-income countries. Though the causal relation of this disparity is beyond the scope of this manuscript, prior studies suggest late presentation and initiation of cancer treatment, barriers to access adequate care, lack of public awareness, limited healthcare facilities, and exposure to common cancer modifiable risk factors as possible reasons for these differences (10, 16).

As previously discussed, many Afghan patients come from provinces that are on the other side of the country, much closer to Iran than to Pakistan. The fact that SKMCH&RC provides free cancer treatment to those who cannot afford to pay, coupled with the fact that it allows unrestricted access to patients from Afghanistan, may explain why patients choose to come to Pakistan. Anecdotally, many patients state that visas for Iran are much harder to obtain than those for Pakistan and, of course, it is very likely that the large number of Afghan patients receiving treatment and attending follow-up appointments at our hospitals have disseminated information about our facilities, and the model on which they operate, within Afghanistan.

We have attempted to highlight here some of the issues faced by patients in Afghanistan in cancer diagnosis and treatment, as well as data related to incidence, tumour types, age at presentation and regional distribution of cancers. Obviously, not all patients suffering from cancer in Afghanistan are able to come to SKMCH&RC for treatment, and there is no accurate estimate of incidence and prevalence of cancer in that country. In this paper, we have tried to draw attention to the difficulties such patients face - as well as the opportunities for treatment that are made available to them – when they choose to seek treatment in Pakistan, as a result of the very limited healthcare resources available within their own country. Their journey is difficult and often dangerous because of ongoing conflict. Cancer care is hard enough at the best of times, but cancer care during times of conflict presents a unique set of challenges. Patients often travel with several relatives, adding to the overall cost as well as causing significant disruption to family life, and to livelihoods.

One reason why cancer care in refugee and migrant populations is under-funded may be the simplistic view that all types of cancer have a uniformly poor prognosis and it is perhaps time to start thinking of the costs of cancer treatment as an investment in the future, rather than simply as a drain on resources.

Educational campaigns to raise public awareness about cancer and cancer prevention, including information on when and where to seek help, should be developed to help detect and treat early-stage cancer which would be less expensive to treat and would also have better outcomes.

We feel our data is important in drawing attention to the possible scale of the problem. Such information ought to be helpful for national health policy planners, as well as for international funding agencies, such as the UNHCR. The nascent efforts to establish cancer services in Afghanistan, which we hope to help with, will hopefully form the nucleus around which such services can develop in the future.

## Data availability statement

The raw data supporting the conclusions of this article will be made available by the authors, upon request.

## Ethics statement

All studies involving human participants are reviewed and approved by Shaukat Khanum Memorial Cancer Hospital and Research Centre, Institutional Review Board (IRB). The IRB reviewed this study and awarded it exempt-status, meaning that individual patient consents did not need to be sought, since this is a retrospective review and no patient-identifiable data are discussed. 

## Author contributions

All authors listed have made a substantial, direct, and intellectual contribution to the work and approved it for publication.
